# Cardiovascular Impact in Patients With COVID-19 in Bangladesh: A Cross-Sectional Study

**DOI:** 10.7759/cureus.98156

**Published:** 2025-11-30

**Authors:** Abed H Khan, Md Mizanur Rahman Khan, Shohael Mahmud Arafat, Elora Sharmin, Lima Asrin Sayami, Sheikh Foyez Ahmed, Chowdhury Adnan Sami

**Affiliations:** 1 Internal Medicine, Bangabandhu Sheikh Mujib Medical University, Dhaka, BGD; 2 Pharmacology and Therapeutics, Bangabandhu Sheikh Mujib Medical University, Dhaka, BGD; 3 Cardiology, National Institute of Cardiovascular Diseases and Hospital, Dhaka, BGD; 4 Cardiology, Bangabandhu Sheikh Mujib Medical University, Dhaka, BGD

**Keywords:** bangladesh, cardiovascular complications, covid-19, hypertension, myocardial infarction

## Abstract

Background

Coronavirus disease 2019 (COVID-19) has been associated with significant cardiovascular complications. While most studies focus on patients with pre-existing cardiac conditions, less is known about the cardiovascular impact in individuals without prior heart disease, particularly in low- and middle-income countries (LMICs). This study aimed to assess the prevalence, clinical profile, and associated factors of new-onset cardiovascular complications among hospitalized COVID-19 patients in Bangladesh without pre-existing heart disease.

Methods

We conducted a cross-sectional observational study at Bangabandhu Sheikh Mujib Medical University (BSMMU) from February to July 2022. Adult patients (≥18 years) hospitalized with reverse transcription-polymerase chain reaction (RT-PCR)-confirmed COVID-19 and no prior cardiovascular history were included. Clinical data, laboratory findings, and electrocardiographic and echocardiographic abnormalities were recorded. Multivariate logistic regression was used to assess associations between demographic variables (age, sex, smoking status) and cardiovascular complications.

Results

Among 182 patients (mean age: 37.5 ± 12.14 years; 52.1% female), new-onset cardiovascular complications were observed in 58 (31.9%) with hypertension, 20 (10.9%) with ischemic heart disease, 19 (10.44%) with myocardial infarction, 16 (8.79%) with heart failure, 11 (6.04%) with arrhythmias, and 10 (5.49%) with cardiomyopathy. Cardiovascular events occurred across all levels of COVID-19 severity, including mild and moderate presentations. No statistically significant associations were found between cardiovascular complications and age, sex, or smoking status.

Conclusions

Cardiovascular complications are common among COVID-19 patients in Bangladesh, even in younger individuals without prior heart disease. These findings highlight the need for proactive cardiac evaluation during and after COVID-19 infection, regardless of baseline cardiovascular risk.

## Introduction

Coronavirus disease 2019 (COVID-19) was declared a pandemic by the World Health Organization (WHO) on March 11, 2020. The disease is caused by severe acute respiratory syndrome coronavirus 2 (SARS-CoV-2), a novel beta-coronavirus that has led to unprecedented global health and economic disruptions. As of August 2021, more than 216 million individuals worldwide had been infected, with over four million deaths reported. Bangladesh has also been significantly affected, recording 1,489,589 confirmed cases and 25,926 deaths by that time [1,2]. The rapid transmission and high morbidity associated with COVID-19 have overwhelmed healthcare systems globally, particularly in resource-limited settings like Bangladesh, where the infrastructure is already strained by both infectious and non-communicable diseases.

COVID-19 manifests with a wide range of clinical symptoms, from mild respiratory illness to severe pneumonia, acute respiratory distress syndrome (ARDS), and multi-organ failure. Despite substantial global research, no specific antiviral therapies have been approved by the U.S. Food and Drug Administration (FDA) for COVID-19 treatment [3]. Current management remains largely supportive, including oxygen therapy, mechanical ventilation, and corticosteroids. Several pharmacologic interventions, such as remdesivir, hydroxychloroquine, chloroquine, azithromycin, corticosteroids, and immunomodulatory agents, have been evaluated, though none have demonstrated definitive efficacy in reducing mortality [4].

Among the numerous complications associated with COVID-19, cardiovascular involvement has emerged as a significant clinical concern. Pre-existing cardiovascular diseases (CVDs), including hypertension, coronary artery disease, and heart failure, have been strongly linked to greater disease severity and increased mortality in COVID-19 patients [5]. This interplay underscores the need for vigilant cardiac monitoring and optimized management of patients with underlying CVD.

Given the substantial burden of CVD and the ongoing challenges posed by COVID-19 in Bangladesh, it is essential to understand the cardiac manifestations of SARS-CoV-2 infection. This study aimed to assess the prevalence, clinical profile, and associated factors of new-onset cardiovascular complications among hospitalized COVID-19 patients in Bangladesh without pre-existing heart disease. By elucidating the cardiovascular impact of SARS-CoV-2 in this population, the study seeks to inform targeted management strategies and improve clinical outcomes.

## Materials and methods

Study design and participants

This was a single-center, cross-sectional observational study conducted at the Cardiology Department of Bangabandhu Sheikh Mujib Medical University (BSMMU), Dhaka, Bangladesh, over a six-month period from February to July 2022. The study included adult patients aged ≥18 years who were admitted with a confirmed diagnosis of COVID-19, as verified by reverse transcription-polymerase chain reaction (RT-PCR) testing for SARS-CoV-2. Inclusion criteria were RT-PCR-confirmed COVID-19 infection and presentation with clinical or investigational features suggestive of myocardial injury, such as chest pain, shortness of breath, electrocardiographic abnormalities, or echocardiographic dysfunction. Importantly, all included patients had no documented history of CVD prior to hospital admission. Patients with COVID-19 were divided into three groups: mild, moderate, and severe COVID-19, according to the WHO.

Mild disease is defined as the presence of symptomatic COVID-19 without evidence of pneumonia or hypoxia. Patients typically present with upper respiratory tract symptoms such as fever, cough, sore throat, nasal congestion, headache, myalgia, or malaise. Importantly, there are no signs of respiratory distress, no requirement for supplemental oxygen, and oxygen saturation (SpO₂) remains within the normal range on room air [6].

Moderate disease is characterized by clinical or radiological evidence of lower respiratory tract involvement consistent with pneumonia, but without the defining features of severe disease. Patients may experience shortness of breath and demonstrate abnormalities on chest imaging; however, they maintain a SpO₂ ≥ 90-94% on room air and do not exhibit signs of severe respiratory distress. This category, therefore, includes pneumonia without hypoxemia or only mild oxygen desaturation [6].

Severe disease is defined by the presence of clinical signs of pneumonia accompanied by at least one of the following: respiratory rate greater than 30 breaths per minute, severe respiratory distress, or oxygen saturation < 90% on room air. Severe COVID-19 is therefore marked by significant hypoxemia, tachypnea, or overt respiratory compromise necessitating oxygen therapy and close monitoring [6].

Exclusion criteria included patients with a history of myocardial infarction within the previous six months, end-stage chronic kidney disease requiring dialysis, or incomplete medical records that precluded cardiovascular assessment.

Data collection

Data were extracted from patient records using a standardized Clinical Record Form (CRF) developed for this study (Appendix 1). Trained data collectors gathered detailed demographic information (age, sex, education, occupation), lifestyle history (smoking status), comorbidities, and clinical severity of COVID-19. Cardiovascular complications were identified based on clinical presentation and diagnostic criteria. Myocardial injury was confirmed via elevated serum cardiac troponin levels. Electrocardiographic (ECG) changes and echocardiographic findings were evaluated to document structural or functional cardiac abnormalities.

The presence of cardiovascular complications, such as hypertension, ischemic heart disease, myocardial infarction, heart failure, arrhythmia, and cardiomyopathy, was documented and categorized as either pre-existing (diagnosed before admission) or newly developed during hospitalization. COVID-19 severity was classified as mild, moderate, or severe based on national guidelines and clinical records.

Ethical approval

This study was approved by the Institutional Review Board (IRB) of Bangabandhu Sheikh Mujib Medical University (BSMMU), Dhaka, Bangladesh (BSMMU/2021/9687). Ethical clearance was granted on 17 September 2021. All procedures were conducted in accordance with the ethical standards of the Helsinki Declaration. Informed written consent was obtained from all participants.

Statistical analysis

All data were entered and analyzed using Microsoft Excel (Microsoft Corporation, Redmond, Washington, United States) and IBM SPSS Statistics for Windows, Version 25 (released 2017; IBM Corp., Armonk, New York, United States). Continuous variables were reported as means with standard deviations (SD), while categorical variables were presented as frequencies and percentages using the n (%) format. Comparative analysis was performed to assess the distribution of cardiovascular complications across age groups, sex, and COVID-19 severity categories. A multivariate logistic regression model was employed to evaluate associations between selected demographic variables (age, sex, smoking history) and the occurrence of cardiovascular complications. Statistical significance was set at a p-value < 0.05.

## Results

Socio-demographic characteristics

A total of 182 patients were included in this study. The mean age was 37.50 ± 12.14 years. Of these, 95 (52.1%) were female, and 87 (47.8%) were male. Most participants, 114 (62.64%), had more than twelve years of education, followed by 37 (20.33%) with 11-12 years, 18 (9.89%) with six to 10 years, and 13 (7.14%) with one to five years of education.

Occupationally, 67 (36.81%) were employed in non-government services, 40 (21.98%) were businessmen, 20 (10.99%) were housewives, 13 (7.14%) were students, 11 (6.04%) were healthcare workers, nine (4.94%) were doctors, 15 (8.24%) worked in government service, and seven (3.85%) were categorized as others.

Regarding smoking history, 100 (54.94%) were former smokers, 62 (34.06%) had never smoked, and 20 (10.99%) were current smokers.

Chronic illnesses were reported by 48 (26.37%) participants, while 134 (73.63%) had no known comorbidities. Among those with chronic conditions, diabetes mellitus was reported in 12 (6.59%), chronic kidney disease in nine (4.94%), hypothyroidism in six (3.29%), COPD in five (2.75%), asthma in four (2.19%), malignancy in two (1.10%), and other conditions in three (1.65%) (Table 1). 

**Table 1:** Socio-demographic characteristics and health status of study participants (n=182) COPD: chronic obstructive airway disease; CKD: chronic kidney disease

Characteristics	Category	Frequency	Percentage (%)
Sex	Female	95	52.1
	Male	87	47.8
Education Level	1-5 years	13	7.14
	6-10 years	18	9.89
	11-12 years	37	20.33
	>12 years	114	62.64
Occupation	Doctor	9	4.94
	Healthcare Worker	11	6.04
	Student	13	7.14
	Housewife	20	10.99
	Businessman	40	21.98
	Government Service	15	8.24
	Non-Government Service	67	36.81
	Others	7	3.85
Smoking History	Never	62	34.06
	Current Smoker	20	10.99
	Quit Smoker	100	54.94
Chronic Diseases	None	134	73.63
	Diabetes	12	6.59
	COPD	5	2.75
	Asthma	4	2.19
	Malignancy	2	1.09
	CKD	9	4.94
	Hypothyroidism	6	3.29
	Others	3	1.65

Cardiovascular complications in COVID-19 patients

A range of cardiovascular complications was identified. The most frequent was hypertension in 58 (31.86%) patients, followed by ischemic heart disease in 20 (10.98%), acute myocardial infarction in 19 (10.44%), heart failure in 16 (8.79%), arrhythmia in 11 (6.04%), and cardiomyopathy in 10 (5.49%) (Table 2). Of these, 25 (13.74%) cases of hypertension, six (3.29%) of ischemic heart disease, nine (4.95%) of acute myocardial infarction, one (0.55%) of heart failure, and six (3.29%) of cardiomyopathy were pre-existing. The remaining cases were newly diagnosed during hospitalization for COVID-19: 33 (18.13%) for hypertension, 14 (7.69%) for ischemic heart disease, 10 (5.49%) for myocardial infarction, 15 (8.24%) for heart failure, 11 (6.04%) for arrhythmia, and four (2.19%) for cardiomyopathy.

**Table 2:** Cardiovascular complications in COVID-19 patients (n=182)

Clinical Manifestation	Frequency	Percentage (%)
Acute Myocardial Infarction (MI)	19	10.44
Heart Failure	16	8.79
Arrhythmia	11	6.04
Cardiomyopathy	10	5.49
Hypertension (HTN)	58	31.86
Ischemic Heart Disease (IHD)	20	10.98

Age and sex distribution of cardiovascular events

Age-stratified analysis revealed that new-onset cardiovascular complications were more prevalent in older age groups. Among patients aged <25 years, nine (42.85%) of 21 developed new cardiovascular system (CVS) complications. In the 26-36 age group, 19 (61.29%) of 31 developed new complications. Among those aged 37-47 years and >48 years, new-onset cardiovascular events occurred in 55 (77.46%) of 71 and 46 (77.97%) of 59, respectively. Across all age groups, the distribution was similar between males and females (Table 3).

**Table 3:** Age and sex distribution of patients with CVS complications *Percentages were calculated within the age groups. CVS: cardiovascular system

Age Group	Gender		CVS Complication Frequency	
	Male (n)	Female (n)	Pre-existing heart disease n (%)^*^	Newly diagnosed n (%)^*^
<25	11	10	1 (4.76)	9 (42.85)
26-36	11	20	1 (3.22)	19 (61.29)
37-47	36	35	2 (1.41)	55 (77.46)
>48	29	30	1 (1.69)	46 (77.97)

Association with COVID-19 severity

Among 134 patients for whom severity data were available, cardiovascular complications occurred across all clinical categories. Hypertension was observed in 35 (26.1%) with mild, 13 (9.7%) with moderate, and 10 (7.5%) with severe COVID-19. Myocardial infarction occurred in seven (5.2%) mild, eight (6.0%) moderate, and four (3.0%) severe cases. Heart failure was present in 10 (7.5%) mild, five (3.7%) moderate, and one (0.7%) severe cases. Arrhythmia was detected in eight (6.0%) mild, two (1.5%) moderate, and one (0.7%) severe cases. Cardiomyopathy occurred in six (4.5%) mild, three (2.2%) moderate, and one (0.7%) severe cases. These findings suggest that cardiovascular involvement is not confined to severe disease alone (Table 4).

**Table 4:** Distribution of CVS complications according to severity CVS: cardiovascular system

CVS Complications	COVID-19 Severity			
	Mild (n)	Moderate (n)	Severe (n)	
Hypertension (HTN)	35	13		10
Ischemic Heart Disease (IHD)	10	6		4
Acute Myocardial Infarction (MI)	7	8		4
Heart Failure	10	5		1
Arrhythmia	8	2		1
Cardiomyopathy	6	3		1

Regression analysis of demographic predictors

Multivariate logistic regression revealed no statistically significant associations between age, sex, or smoking status and the development of cardiovascular complications. Age (β = 0.0214, p = 0.316), sex (β = 0.5506, p = 0.221), and smoking (β = -0.2018, p = 0.411) were not significant predictors. The 95% confidence intervals for each variable included the null, indicating no independent association. These findings indicate that in this cohort, traditional cardiovascular risk factors did not significantly influence the likelihood of developing cardiovascular complications during COVID-19 (Table 5).

**Table 5:** Multivariate logistic regression analysis of predictors of cardiovascular complications

Predictor Variable	Coefficient (β)	95% Confidence Interval	Standard Error	z-value	p-value
Age	0.0214	–0.020 to 0.063	0.021	1.002	0.316
Sex	0.5506	–0.331 to 1.432	0.450	1.225	0.221
Smoking Status	–0.2018	–0.683 to 0.279	0.245	–0.822	0.411

## Discussion

In this cohort of 182 hospitalized COVID-19 patients in Bangladesh with no prior CVD, we observed a substantial incidence of new cardiovascular complications. Notably, 31.9% developed new-onset hypertension, 10.4% experienced acute myocardial infarction, 10.9% developed ischemic heart disease, 8.8% developed heart failure, 6% had new arrhythmias, and 5.5% developed cardiomyopathy. These events occurred across all degrees of COVID-19 severity, including mild and moderate cases. Such findings underscore that even relatively young, previously healthy individuals are vulnerable to COVID-19-related cardiovascular manifestations. Indeed, our patients’ median age was lower than that of many Western cohorts, reflecting Bangladesh’s younger population (median age 26.7 years, with only 7% over 65) [7]. Despite this demographic advantage, COVID-19 had a significant cardiovascular impact in our cohort, consistent with reports that the virus can directly affect the cardiovascular system even in younger populations [8].

Our results align with and extend existing literature on COVID-19’s cardiac complications. Global studies have documented a broad spectrum of acute COVID-19 cardiovascular injuries, including myocardial injury, acute coronary syndromes, arrhythmias, myocarditis, and heart failure [9]. A meta-analysis of studies early in the pandemic reported incidences of myocardial injury in ~21% and arrhythmias in ~15% of hospitalized patients [9]. Our observed 6% arrhythmia rate is lower than those early estimates, likely reflecting our cohort’s younger age and inclusion of less severe cases. Reported arrhythmia frequencies in COVID-19 have varied widely, from ~0.1% up to ~8% for ventricular arrhythmias, depending on disease severity [10], with one study noting arrhythmias in 19.6% of hospitalized patients [11]. Thus, our findings fall within the expected range, and they reinforce that even in non-critically ill patients, arrhythmic complications can occur. Importantly, our multivariable analysis found no significant association between patient age, sex, or smoking status and the risk of developing cardiovascular complications. This lack of a clear predictor suggests that the direct viral and inflammatory effects of SARS-CoV-2 may override traditional risk factors in precipitating acute cardiac events, a notion supported by case reports of young, low-risk patients experiencing COVID-19 myocarditis or acute coronary syndromes [8].

The incidence of acute ischemic events in our study is noteworthy. We found ~10% of patients had a myocardial infarction during hospitalization, which is higher than some early reports (a 2021 systematic review noted acute coronary syndromes in only ~1% of COVID-19 patients [9]) yet similar to rates observed in South Asian cohorts. For example, an Indian hospital study reported 9.2% of COVID-19 inpatients presented with acute coronary syndrome, including ST-elevation and non-ST-elevation myocardial infarction [12]. COVID-19 can provoke plaque rupture or thrombosis via systemic inflammation and coagulopathy, explaining these ischemic presentations. Our finding that 5.5% developed a new cardiomyopathy (which may encompass stress cardiomyopathy or myocarditis) is also in line with global observations of COVID-19’s myocardial involvement. A French multicenter study noted that patients under 45 had higher rates of myocarditis and pericarditis compared to older patients [8], indicating a propensity for COVID-19 to trigger myocardial inflammation in younger hosts. Direct myocardial infection, cytokine surge, and microvascular thromboses are proposed mechanisms for these syndromes [9].

One of the most striking outcomes in our cohort was the 31.9% incidence of new-onset hypertension. None of the patients had known hypertension prior to COVID-19, yet nearly one-third were diagnosed with elevated blood pressure during their illness. Transient stress, sympathetic activation, corticosteroid therapy, and disruption of the angiotensin-converting enzyme 2 (ACE2) pathway by SARS-CoV-2 are potential contributors to acute blood pressure elevation. Our finding is higher than what has been reported in some larger studies, but comparable data underscore that COVID-19 can precipitate persistent hypertension. For instance, in a cohort of over 45,000 patients in New York, 21.0% of hospitalized COVID-19 patients with no history of hypertension developed hypertension by the time of discharge [13], and about 20.6% remained hypertensive at six-month follow-up [13]. These convergent findings raise concern that COVID-19 may lead to incident hypertension, possibly permanent, thereby enlarging the future pool of individuals at risk for cardiovascular events. From a public health perspective, this is especially important for countries like Bangladesh, where the baseline prevalence of hypertension was ~20% in adults pre-pandemic [14]. An influx of new hypertensive patients post-COVID-19 could accelerate the ongoing rise of CVD in these populations. Continuous surveillance is needed to determine whether post-COVID hypertension abates or requires long-term management.

Clinical and public health implications

The demonstration of frequent cardiovascular complications in a young, previously healthy cohort and across all severities of COVID-19 has several implications. Clinicians should maintain a high index of suspicion for cardiac involvement in any COVID-19 patient, not only in the elderly or those with pre-existing heart disease. Even patients with mild to moderate acute illness may develop latent complications such as silent myocardial injury, arrhythmia, or subclinical ventricular dysfunction. Our findings support a proactive approach to cardiovascular evaluation in COVID-19 care. During acute infection, monitoring of cardiac biomarkers (troponin, B-type natriuretic peptide (BNP)), electrocardiography, and echocardiography (as indicated by symptoms) should be considered, even in patients without prior CVD. Early identification of myocarditis, acute coronary syndrome, or arrhythmia allows for timely treatment (e.g., coronary intervention, arrhythmia management, or cardioprotective therapies) that could improve outcomes.

Equally important is the post-recovery follow-up. Growing evidence indicates that COVID-19 survivors face elevated risks of cardiovascular events for months after the acute phase. A comprehensive U.S. Veterans Affairs study showed that at one year post-infection, COVID-19 survivors had increased incidence of a range of cardiovascular conditions, including arrhythmias, ischemic heart disease, myocarditis, heart failure, and thromboembolism, even among those who had mild initial illness [15]. Our results mirror this global finding and reinforce that surveillance should extend beyond the resolution of acute COVID-19. Patients should be educated about symptoms of potential cardiac complications (chest pain, palpitations, dyspnea, syncope) and advised to seek evaluation if these occur after recovery. From a health systems standpoint, setting up post-COVID clinics or follow-up protocols that include cardiovascular screening would be prudent. For example, guidelines now suggest that patients with persistent symptoms or high-risk features undergo targeted workups, such as blood pressure monitoring, electrocardiograms, ambulatory rhythm monitoring for palpitations, echocardiography for those with dyspnea or elevated biomarkers, and cardiac MRI if myocarditis is suspected. Such strategies align with the recommendations of expert consensus panels and scientific statements, which call for vigilance in detecting COVID-19’s cardiovascular sequelae.

In Bangladesh, where the healthcare infrastructure is already managing a dual burden of infectious diseases and rising non-communicable diseases, our findings highlight a new dimension to the pandemic’s impact. Younger patients who were not previously on the radar for CVD may now need cardiovascular care. This calls for strengthening interdisciplinary collaboration between infectious disease specialists, internists, and cardiologists. Follow-up clinics for recovered COVID-19 patients could implement routine cardiovascular risk assessment, including measuring blood pressure, glucose, and lipid levels, since COVID-19 may unmask underlying cardiometabolic issues. In particular, given the high rate of new-onset hypertension we observed, it would be sensible to recheck blood pressure in convalescent COVID-19 patients and initiate treatment or lifestyle counseling if hypertension persists. Early management of post-COVID hypertension or arrhythmias could reduce the long-term burden of stroke, heart failure, or other complications in this population.

Finally, our study underscores that the pandemic’s toll extends beyond acute infections and deaths; it includes a wave of cardiovascular morbidity that could strain healthcare systems in the future. Public health authorities should incorporate these insights into planning and resource allocation. This may involve creating awareness programs about heart health in post-COVID patients and ensuring access to appropriate diagnostic tests (such as ECGs and echocardiography) and specialty care. It also strengthens the case for COVID-19 prevention (vaccination and early treatment), as preventing infections may also avert the downstream cardiovascular complications. Our findings contribute to the growing recognition that COVID-19 is not solely a respiratory illness but a systemic disease with diverse long-term effects. Routine ECG and cardiac biomarker screening should be considered even for COVID-19 patients without prior CVD.

Limitations

This study has several limitations. First, as a single-center investigation conducted at a tertiary care hospital in Bangladesh, the findings may not be generalizable to broader populations or healthcare settings. Second, the cross-sectional design precludes establishing causal relationships between SARS-CoV-2 infection and the observed cardiovascular complications. Third, the absence of a control group (e.g., non-COVID-19 patients or those with prior CVD) limits the ability to determine the relative risk directly attributable to COVID-19. Fourth, not all participants underwent comprehensive cardiac biomarker testing or advanced imaging such as cardiac MRI or coronary angiography, which may have led to underdiagnosis of subclinical myocardial injury or myocarditis. Fifth, although individuals with known cardiac disease were excluded, undiagnosed or subclinical pre-existing conditions may have been missed due to limitations in baseline screening. Also, we did not assess the persistence of hypertension post-discharge. A key limitation of this study is the absence of several important confounding variables, such as diabetes mellitus, chronic kidney disease, body mass index, steroid exposure, and COVID-19 severity, in the regression analysis. These factors are known to influence cardiovascular outcomes and may have contributed to residual confounding. Finally, as outcomes were assessed only during hospitalization, the study does not capture long-term cardiovascular sequelae following recovery from COVID-19, which may be substantial based on emerging global evidence.

## Conclusions

Cardiovascular complications, including hypertension, myocardial infarction, and arrhythmias, were frequently observed in young, previously healthy COVID-19 patients in this Bangladeshi cohort. These findings highlight that cardiovascular involvement can occur even in mild to moderate cases and in the absence of pre-existing heart disease. The lack of association with traditional risk factors underscores the systemic nature of SARS-CoV-2 infection. This study adds novel evidence from a low-income setting and calls for routine cardiovascular screening and long-term follow-up in COVID-19 survivors, particularly in South Asian populations with an evolving burden of non-communicable diseases. Early recognition through ECG and cardiac biomarker screening is recommended, and larger longitudinal studies are warranted.

## Figures and Tables

**Figure 1:**
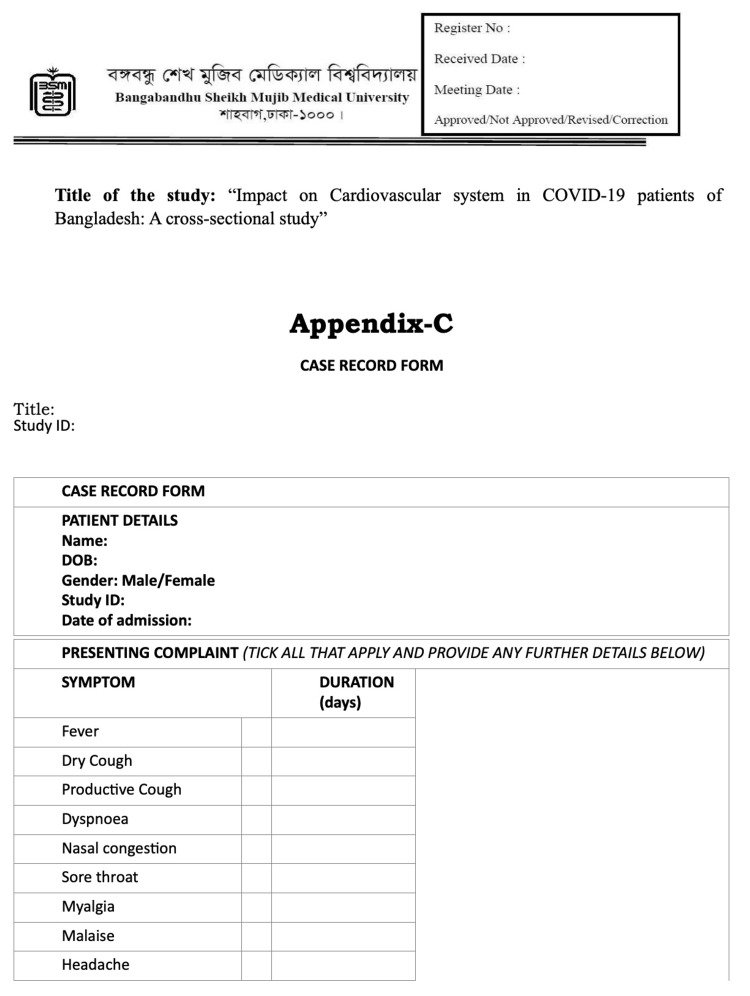
Clinical Record Form (Page 1)

**Figure 2:**
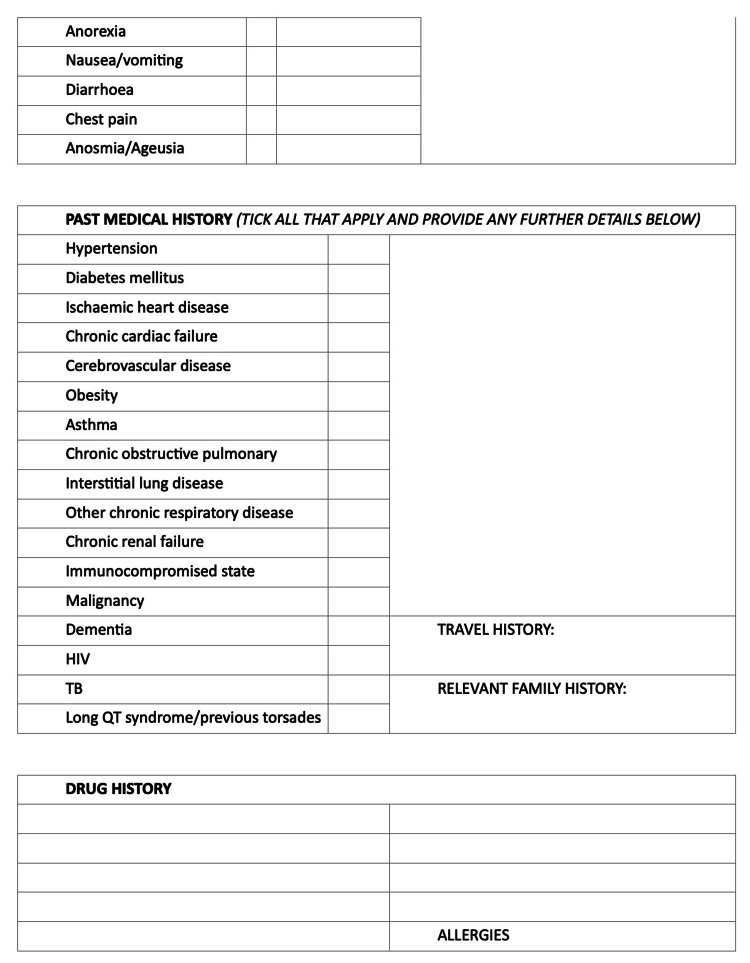
Clinical Record Form (Page 2)

**Figure 3:**
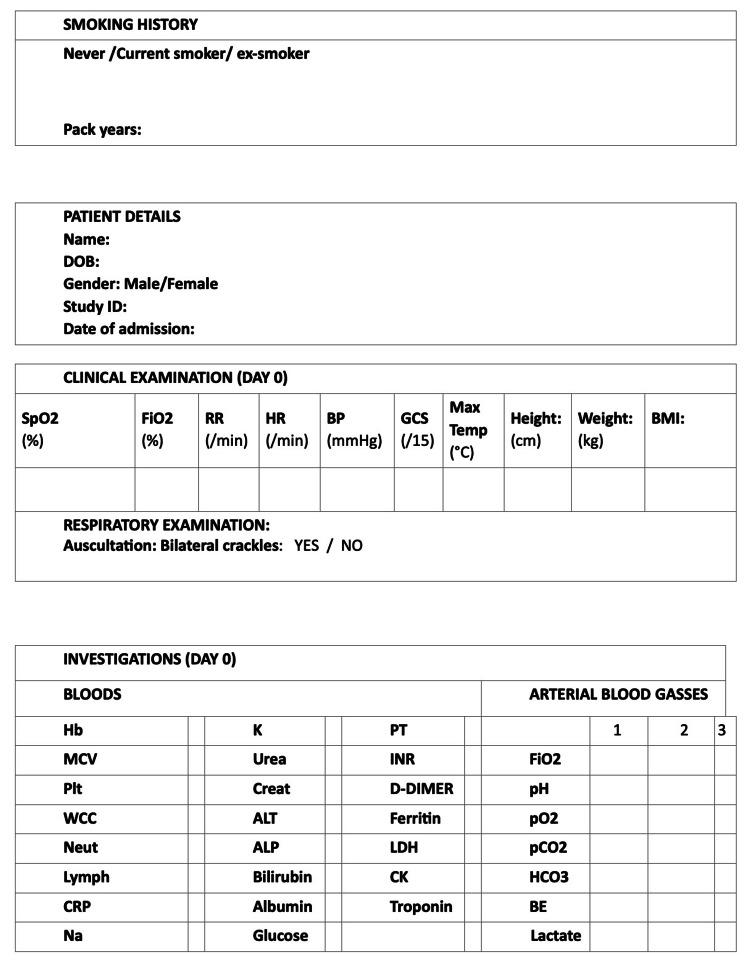
Clinical Record Form (Page 3)

**Figure 4:**
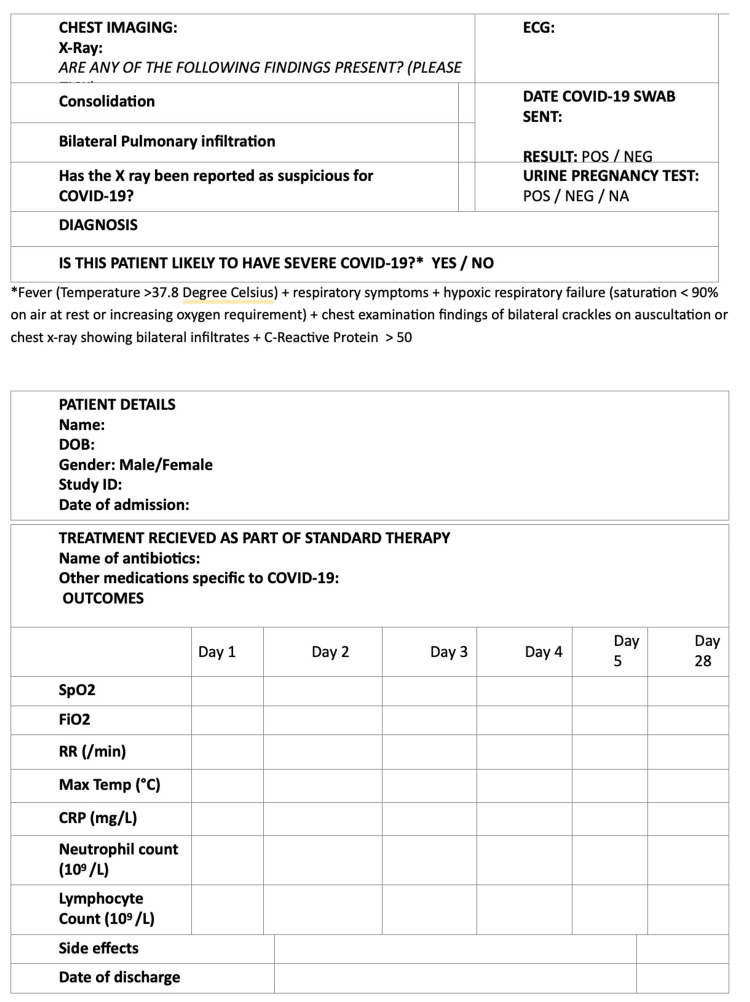
Clinical Record Form (Page 4)

**Figure 5:**
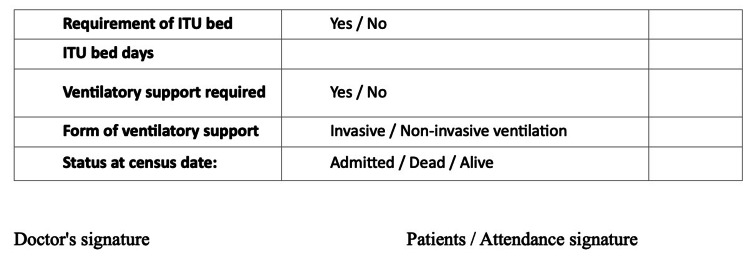
Clinical Record Form (Page 5)

## Appendices

Appendix 1
